# Bioinspired Robust Gas‐Permeable On‐Skin Electronics: Armor‐Designed Nanoporous Flash Graphene Assembly Enhancing Mechanical Resilience

**DOI:** 10.1002/advs.202402759

**Published:** 2024-05-05

**Authors:** Yang Chen, Zixuan Liu, Zhigang Wang, Ying Yi, Chunjie Yan, Wenxia Xu, Feng Zhou, Yuting Gao, Qitao Zhou, Cheng Zhang, Heng Deng

**Affiliations:** ^1^ Faculty of Materials Science and Chemistry China University of Geosciences Wuhan 430074 P. R. China; ^2^ College of Engineering Nanjing Agricultural University Nanjing 210031 P. R. China; ^3^ School of Mechanical Engineering and Electronic Information China University of Geosciences Wuhan 430074 P. R. China; ^4^ Shenzhen Research Institute China University of Geosciences Shenzhen 518000 P. R. China

**Keywords:** flash graphene, gas‐permeable, human‐machine interaction, soft electrode, triboelectric nanogenerator sensor

## Abstract

Soft on‐skin electrodes play an important role in wearable technologies, requiring attributes such as wearing comfort, high conductivity, and gas permeability. However, conventional fabrication methods often compromise simplicity, cost‐effectiveness, or mechanical resilience. In this study, a mechanically robust and gas‐permeable on‐skin electrode is presented that incorporates Flash Graphene (FG) integrated with a bioinspired armor design. FG, synthesized through Flash Joule Heating process, offers a small‐sized and turbostratic arrangement that is ideal for the assembly of a conductive network with nanopore structures. Screen‐printing is used to embed the FG assembly into the framework of polypropylene melt‐blown nonwoven fabrics (PPMF), forming a soft on‐skin electrode with low sheet resistance (125.2 ± 4.7 Ω/□) and high gas permeability (≈10.08 mg cm⁻^2^ h⁻¹). The “armor” framework ensures enduring mechanical stability through adhesion, washability, and 10,000 cycles of mechanical contact friction tests. Demonstrating capabilities in electrocardiogram (ECG) and electromyogram (EMG) monitoring, along with serving as a self‐powered triboelectric sensor, the FG/PPMF electrode holds promise for scalable, high‐performance flexible sensing applications, thereby enriching the landscape of integrated wearable technologies.

## Graphical abstract

This work presents a mechanically robust, gas‐permeable on‐skin electrode integrating Flash Graphene (FG) with a bioinspired armor design. FG's turbostratic property facilitates nanoporous assembly within a polypropylene melt‐blown nonwoven fabrics (PPMF) substrate, ensuring high gas permeability. The PPMF outer layer acts as protective “armor” for FG, promising applications in wearable sensing, human body monitoring, and human‐computer interaction.

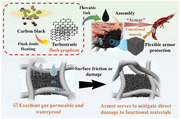

## 1 Introduction

Soft on‐skin electrodes have prominently emerged in recent decades, driven by their potential contributions to pivotal domains such as motion detection, energy storage, human‐computer interaction, and medical monitoring.^[^ ^1^, ^2^, ^3^, ^4^, ^5^ ^]^ Distinguished by attributes such as wearing comfort, high conductivity, lightweight properties, and user‐friendly operation, this class of materials is well‐suited for monitoring physiological signals, exemplified by electromyography (EMG) and electrocardiography (ECG).^[^ ^6^, ^7^ ^]^ Furthermore, their versatility extends to serving as effective components in wearable triboelectric nanogenerators (TENGs), significantly broadening their utility in energy harvesting.^[^ ^8^, ^9^, ^10^ ^]^ To expand the applications of on‐skin electrodes, gas permeability is a critical consideration for wearable comfort. As on‐skin electronics, the electrodes will be in direct contact with human skin. The absence of adequate gas permeability may lead to discomfort through moisture and heat accumulation, potentially causing prolonged skin abrasion, itching, and inflammation.^[^ ^11^, ^12^, ^13^ ^]^ Therefore, the development of gas‐permeable soft electrodes has become a critical pursuit. Moreover, the incorporation of such gas permeability is not only a functional necessity but also a fundamental characteristic defining usability and effectiveness in on‐skin devices.^[^ ^3^ ^]^

In the pursuit of fabricating gas‐permeable soft on‐skin electrodes, the common strategy involves integrating a porous, soft substrate with conductive active materials. This approach aims to synergize the gas permeability of the porous substrate with the highly conductive functionalities of the active materials.^[^ ^14^ ^]^ To preserve optimal gas permeability, one method entails conformally covering the porous substrate's structure with a thin layer of noble metals like gold or silver.^[^ ^15^, ^16^, ^17^, ^18^ ^]^ Such conformal coating ensures the maintenance of the porous architecture. However, this method often involves costly and complex processes such as e‐beam or photolithography, sputtering, and thermal deposition, which limits its practical applicability.^[^ ^19^ ^]^ Alternatively, a more straightforward approach involves laminating the porous substrate with a gas‐permeable conductive layer using filtering deposition. This layer can be made by conductive nano‐wires, porous laser‐induced graphene, or liquid metal, offering simplicity while maintaining high conductivity and gas permeability.^[^ ^6^, ^10^, ^20^, ^21^, ^22^, ^23^, ^24^, ^25^, ^26^ ^]^ Nevertheless, this method exposes the conductive layer on the substrate's surface, posing a challenge as repeated contact with human skin or cloth textures may damage the exposed conductive materials, diminishing their conductivity. Consequently, the development of a simple method for fabricating mechanical rubout and gas‐permeable on‐skin electrodes remains a significant challenge. And the primary concern in fabricating such mechanical rubout and gas‐permeable on‐skin electrodes remains how to protect the fragile functional and conductive nanomaterials.

For protective purposes, natural creatures have evolved armors or exoskeletons to shield their vulnerable and functional main bodies effectively. For example, crayfish or pangolins have developed flexible and robust armor, providing effective protection for their functional muscles while flexing with the muscles during their movements. Such an armored design has inspired researchers to construct mechanically resilient composite functional materials. For instance, the armored design strategy has proven effective in creating robust superhydrophobic surfaces,^[^ ^27^, ^28^ ^]^ where functional nanofillers are embedded within a micro framework for protection against external mechanical contact or friction. Translating this bioinspired armored design concept, it could be adopted to fabricate robust gas‐permeable on‐skin electronics by embedding gas‐permeable conductive fillers inside the protective framework. In this design, the gas‐permeable conductive fillers will be shielded by the porous framework from external mechanical forces while maintaining their electrical conductivity and gas permeability. The challenge lies in finding a filler with high conductivity and gas permeability. In this context, flash graphene (FG), synthesized through the cost‐effective Flash Joule Heating (FJH) process,^[^ ^29^ ^]^ emerges as a suitable candidate. During the FJH process, high reaction temperatures (over 3000 k) can be achieved in a short time of several microseconds. Due to such non‐equilibrium reaction conditions, small‐sized and turbostratic FG flakes are obtained.^[^ ^29^, ^30^ ^]^ The small size (≈30 nm) of FG makes it easy to penetrate into the porous structure of the protective framework. The turbostratic feature prevents AB‐stacking of graphene flakes, facilitating their assembly into a continuous network but with nanopore structures. This assembly ensures a conductive pathway while maintaining gas‐permeability, which can result in a highly conductive, mechanically robust, and gas‐permeable on‐skin electrode.

**Figure 1:**
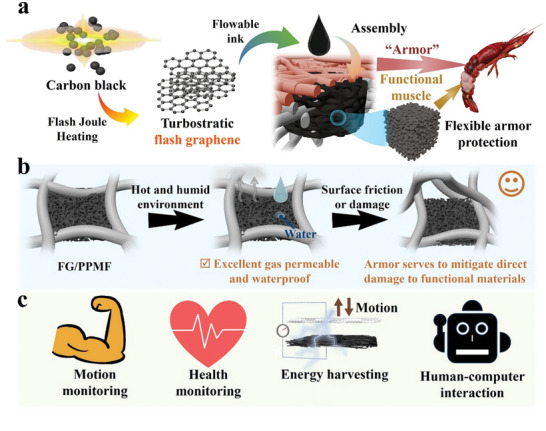
Design, structure, and functions of the FG/PPMF electrodes with excellent mechanical robust and gas permeability. a) The “armor” strategy is designed for FG/PPMF electrodes, with PPMF serving as a protective layer for the internal FG assembly, forming a porous conductive network. It resembles a crayfish shell safeguarding crayfish muscle. The turbostratic nature of FG contributes to the formation of this porous structure. FG is assembled in situ in the form of ink deep inside PPMF to create FG/PPMF. b) FG/PPMF benefits from protection by the surrounding PPMF, shielding the conductive functional materials from direct external forces. It also exhibits excellent gas permeability and waterproofing. c) FG/PPMF with significant potential in the realms of human monitoring and human‐computer interaction.

In our work, we delineate the innovative approach of utilizing polypropylene melt‐Blown nonwoven fabrics (PPMF) as a form of flexible armor to protect the gas‐permeable FG assembly within the matrix (**Figure** **1**a), elucidating the intricacies of our flexible armor design. The PPMF, analogous to crayfish shell, envelops and safeguards the FG, serving as a barrier against direct external forces (Figure 1b). This protective mechanism is integral in preserving the structural integrity and robustness of the FG/PPMF electrode. The porous substrate is completely filled with FG gas‐permeable filler, imparting the electrodes with not only low sheet resistance but also fulfilling the characteristics of high gas permeability for long‐term wear on the human body. The resulting soft electrode exhibited high conductivity with a low sheet resistance of 125.2 ± 4.7 Ω/□. Due to the nanopore of the FG assembly, the soft electrode demonstrated exceptional gas permeability (≈10.08 mg cm^−2^ h^−1^), retaining 98% gas permeability compared to pristine PPMF. The armor design rendered the soft electrode mechanically robust, maintaining electrical stability even after adhesion tests, washable tests, and 10000 cycles of mechanical contact friction tests. As demonstrations, the soft electrode was adopted as an on‐skin sensor for monitoring ECG and EMG signals and a wearable TENG based self‐powered sensor for real‐time monitoring of human activities (Figure 1c). These demonstrations indicate the huge potential of the developed on‐skin electrodes in emerging sensing applications and future human‐machine interaction scenarios.

## 2 Results and Discussion

**Figure 2:**
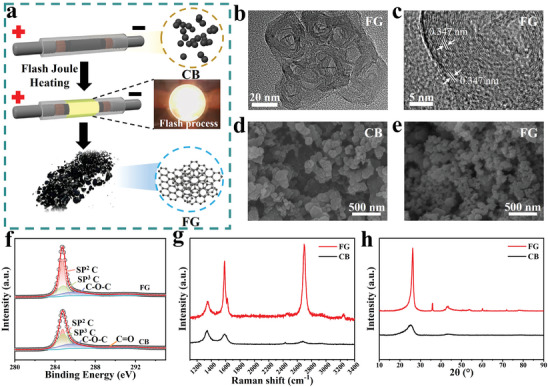
Preparation and characterization of FG. a) Schematic representation of the production of FG by FJH process, utilizing CB as the source material. b,c) HR‐TEM image of FG in different magnifications. d,e) SEM image of CB (d) and FG (e). f) XPS C 1 s spectra, g) Raman spectra, and h) XRD patterns of CB and FG.

The FJH technique was employed to fabricate flash graphene (FG) using carbon black (CB) as the starting material, as illustrated in **Figure** **2**a. Following the supply of a high current to CB through ten parallel capacitors, a notable and intense flash of light becomes observable (Figure S1 and Video S1, Supporting Information). According to previous literature, the peak temperature of the reaction can easily reach 3000 K, transforming the CB into FG.^[^ ^29^ ^]^ The resulting FG exhibited hexagonal flask morphologies of ≈ 30 nm in length, as depicted in the transmission electron microscopy (TEM) images (Figure 2b). High‐resolution TEM revealed an interatomic spacing of 0.347 nm, confirming the formation of graphene (Figure 2c; Figure S2, Supporting Information).^[^ ^31^ ^]^ Notably, the FG exhibited a significantly smaller size compared to the original CB (Figure 2d,e), attributed to the evaporation of heteroatom content during the high‐temperature FJH treatment. X‐ray photoelectron spectroscopy (XPS) analysis can support this, wherein the FG reveals much lower heteroatom content compared to CB (Figure 2f). The disappearance of the C ═ O bond and the sublimation of O elements also signify the impact of the high‐temperature treatment. Additionally, the XPS profile features a distinct peak at 284.68 eV, corresponding to the C ═ C bond of graphene, confirming graphene formation.

Importantly, the non‐equilibrium FJH reaction, characterized by rapid heating and cooling rates, prevented graphene layer stacking, resulting in turbostratic graphene. The Raman and X‐ray diffraction (XRD) spectra can provide crucial insights into the turbostratic feature of flash graphene (FG). As shown in Figure 2g, the Raman spectrum exhibited characteristic peaks at 1350, 1581, and 2694 cm^−1^ corresponding to the D, G, and 2D bands, with a 2D/G ratio of ≈1.23 indicative of high‐quality graphene generation.^[^ ^29^ ^]^ The additional peaks TS_1_ and TS_2_ at ≈1700 cm^−1^ to ≈2100 cm^−1^ confirmed the turbostratic nature of FG, and the M‐peak of absence indicates a lack of AB stacking (Figure S3, Supporting Information).^[^ ^32^, ^33^ ^]^ The XRD analysis revealed a well‐defined (002) peak, signifying the successful graphitization of CB particles. Furthermore, an asymmetric (002) peak and a weak (100) peak emphasized the turbostratic structure of FG (Figure 2h).^[^ ^30^ ^]^

**Figure 3:**
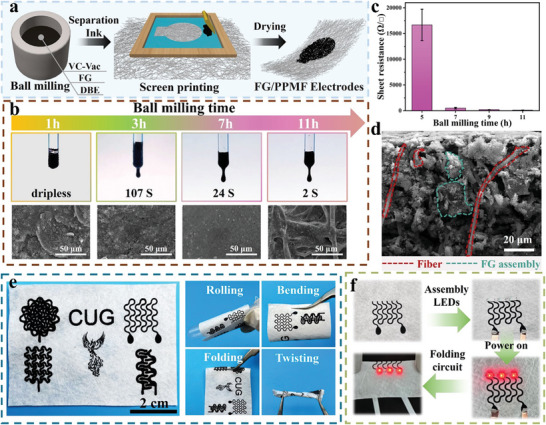
Preparation of screen‐printed FG/PPMF electrodes and optimization of ball milling time. a) Procedure for the preparation of FG/PPMF electrodes through screen printing. b) Flow characteristics of FG ink with various ball milling times and the corresponding surface micromorphology of PPMF printed with these FG inks. c) Sheet resistance of FG/PPMF electrodes corresponding to different ball milling times. d) Cross‐sectional SEM image of FG PPMF electrode showing the in‐depth penetration of FG assembly into the pores of PPMF farmwork. e) Optical images of the screen‐printed flexible FG/PPMF electrodes. f) Demonstration of screen‐printed FG serpentine circuit used to light LEDs under mechanical folding.

In order to fabricate FG‐based soft electrodes through the armor design strategy, FG was initially prepared as a conductive ink and then permeated and filled inside the pore structure of PPMF via screen printing, as illustrated in **Figure** **3**a. The chosen commercial PPMF, which is often used in medical masks, served as an ideal substrate for the soft porous framework materials (Figure S4, Supporting Information) due to its flexibility, lightweight, low cost, and excellent mechanical properties.^[^ ^34^ ^]^ It should be noted that, in addition to the commercial PPMF, sterilized PPMF from waste medical masks could also be utilized in the fabrication of soft electrodes, demonstrating an eco‐friendly approach to repurposing discarded medical waste (Figure S5, Supporting Information). To formulate the FG conductive ink, FG was ball‐milled with the polymer binder Vinyl chloride‐vinyl acetate copolymer (VC‐Vac) and the solvent dimethyl mixed dibasic acid ester (DBE).^[^ ^35^ ^]^ Due to the turbostratic arrangement of FG, it could be well dispersed in DBE (Figure S6, Supporting Information), ensuring the ink's long‐term stability and good flowability, thereby facilitating the smooth penetration of ink into the porous fabric framework during screen printing. During the ball milling process, the thorough mixing of FG and the polymer binder was essential. With extended ball milling time, the ink flowability could be notably enhanced (Figure 3b; Figure S7a, Supporting Information). Moreover, the impact of ball milling time on ink properties was evident in the sheet resistance of printed patterns, with longer durations ensuring the formation of a continuous conductive network (Figure 3c). As a result, an 11‐hour ball milling duration proved optimal, allowing the ink to penetrate the porous fabric framework effectively, as demonstrated in the cross‐sectional SEM image of the FG/PPMF electrode (Figure 3d). The resulting soft electrode exhibited good electrical conductivity, with a low sheet resistance of 125.2 ± 4.7 Ω/□.

In addition to ball milling time, we assessed the influence of ink solids content on the printing process, identifying a 25.5% solids content as optimal for screen printing accurate patterns with a resolution as low as 500 µm (Figures S7b and Figure S8, Supporting Information). Using the homemade screen‐printing device, FG could be printed into different sensor patterns and integrated on the same PPMF substrate. These sensor patterns can be mechanically deformed, such as bending, rolling, folding, and twisting deformation (Figure 3e). The results show that the sensors possess reliable flexibility and wearability, demonstrating the potential of on‐skin or wearable devices. Additionally, we demonstrated the potential of FG ink in flexible printed circuits. As shown in Figure 3f and Video S2 (Supporting Information), a serpentine circuit with patterned gaps to connect three LEDs was printed. By applying a 9 V voltage, the LEDs could be lit up stably even under the condition of mechanical folding. To verify the conductive stability under extreme deformation, we fabricated a long FG/PPMF electrode to light up three LEDs (Figure S9, Supporting Information). Even under extreme twisting deformation, the conductivity of the circuit remained stable, suggesting the FG/PPMF electrode's ability to work reliably in complex external environmental changes.

**Figure 4:**
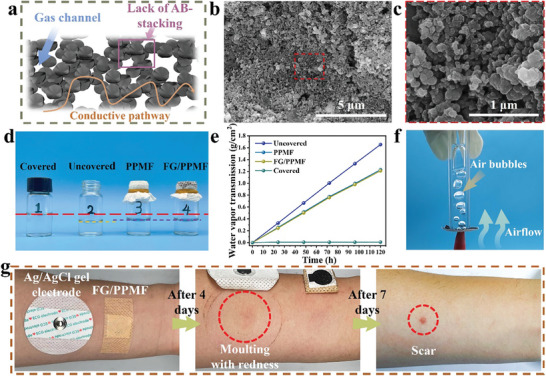
Gas permeability and long‐term wear comfort testing of FG/PPMF electrodes. a) Schematic illustration of the gas‐permeability mechanism of FG/PPMF electrodes, which can be attributed to the turbostratic arrangement of FG. b‐c) SEM images of FG assembly in FG/PPMF electrode. d) Experimental setup for measuring water vapor permeability. e) Water‐vapor transmission of covered, uncovered, PPMF, and FG/PPMF as a function of elapsed time. f) Photograph demonstrates the gas permeability of FG/PPMF. g) Optical images showing the skin condition of the forearm of a volunteer covered with the Ag/AgCl gel electrode and the FG/PPMF electrode.

In the realm of on‐skin applications, electrodes possessing high gas permeability are highly desirable. This attribute facilitates the rapid evaporation of moisture and sweat from the skin, mitigating discomfort and reducing the risk of inflammation. In the FG/PPMF electrode structure, the micro pores within the PPMF substrate were nearly completely filled with FG fillers (Figure 3d). The incorporation of FG seems to obstruct the microscopic porous structure of PPMF, which could potentially reduce its gas permeability. However, owing to the turbostratic characteristics of FG, it induces a disordered arrangement, thereby preventing dense packing (**Figure** **4**a). Consequently, the FG assembly exhibits plentiful nanopores ranging from 200 to 400 nm (Figure 4b,c), ensuring an abundant presence of permeable channels while maintaining the conductive pathway (Figure 4a). To quantify the gas permeability, the water vapor transport rate at 35 °C was estimated by monitoring the weight loss of water over five days. As shown in Figure 4d,e the uncovered control group exhibited an average water vapor transport rate of ≈13.77 mg cm^−2^ h^−1^. The PPMF, used as a liner in masks with high gas permeability, demonstrated good gas permeability with a rate of ≈10.23 mg cm^−2^ h^−1^. Remarkably, the FG/PPMF sample exhibited almost the same water vapor transport rate (≈98%) as the pure PPMF, affirming the highly gas‐permeable nature of the FG assembly. The effective gas permeability was further demonstrated through air‐blowing experiments. As shown in Figure 4f and Video S3 (Supporting Information), an FG/PPMF film was placed between water and air. Upon introducing air into the FG/PPMF using a rubber suction bulb, a significant presence of air bubbles emerged in the water. This observation provides evidence confirming the exceptional gas permeability of FG/PPMF. The high gas permeability of FG/PPMF electrodes will offer long‐term wearing comfort by allowing the rapid evaporation of moisture and sweat from the skin.

Skin electrodes intended for long‐term wear must demonstrate good biocompatibility. Therefore, we characterized the biocompatibility of FG/PPMF. After 24 hours of co‐culturing FG/PPMF with L929 cells, we observed that the growth status of the cells was similar to that of the control group, and the presence of dead L929 cells was scarcely observable in the microscope images (Figure S10, Supporting Information). Based on these findings, we concluded that FG/PPMF is safe and suitable for application as a wearable device for humans. Furthermore, we conducted a comparative study to verify the comfort of the electrodes during prolonged wear, volunteers wore non‐breathable commercial Ag/AgCl gel electrodes on their forearm skin for four days, leading to visible skin peeling, redness, and the formation of scars that persisted for over seven days without complete healing. Contrarily, the highly gas‐permeable FG/PPMF electrode exhibited exceptional skin compatibility, with the volunteer wearing them for the same duration without any signs of discomfort (Figure 4g).

**Figure 5:**
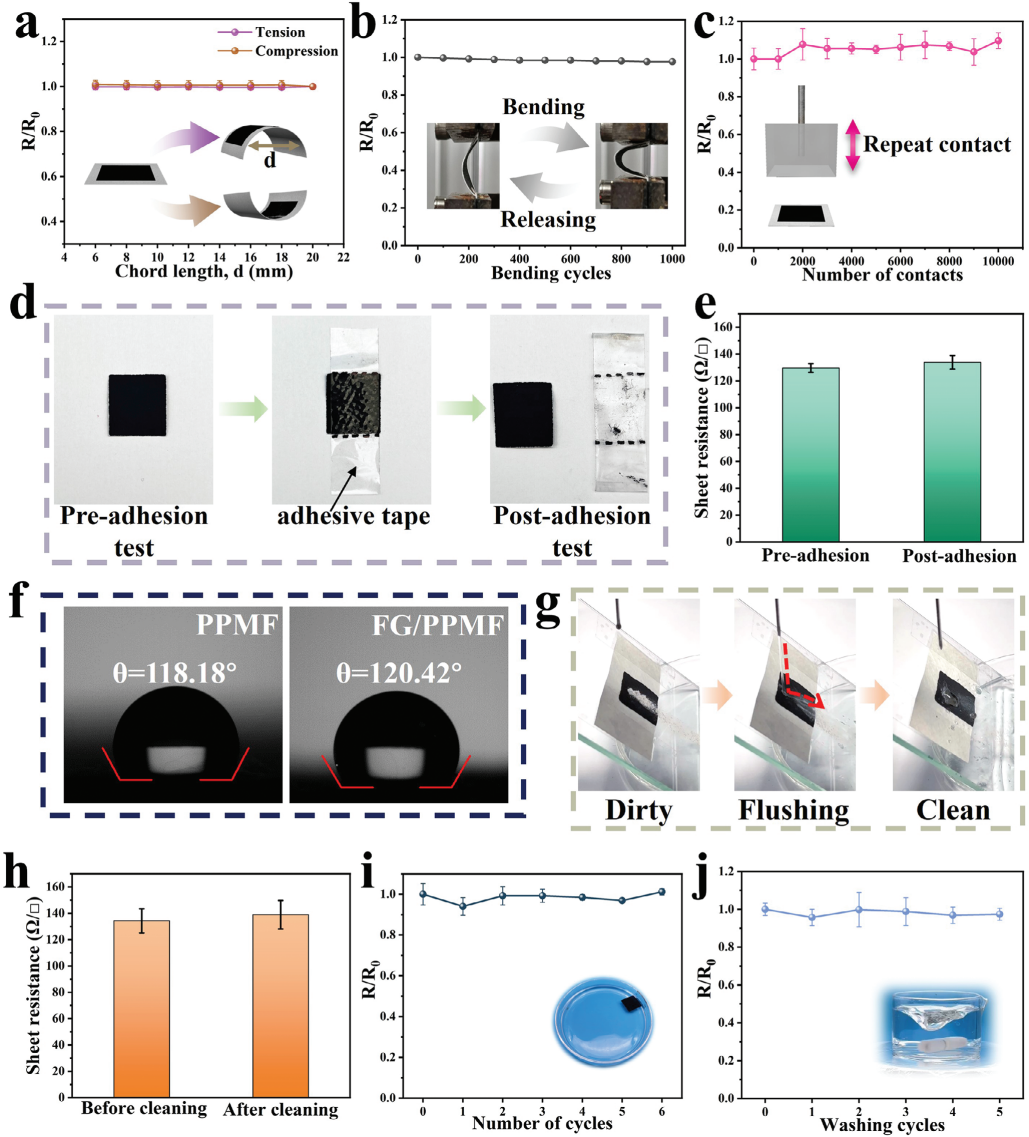
Mechanical stability and waterproofing property of the FG/PPMF electrode. a) Relative resistance variation of the FG/PPMF electrode with variable degrees of bending. b) Relative resistance variation of the FG/PPMF electrode over 1000 bending cycles. c) Relative resistance variation of the FG/PPMF electrode over 10 000 mechanical contact cycles. d) Images depicting the adhesion test procedure for the FG/PPMF electrode using adhesive tape. e) Sheet resistance of the FG/PPMF electrode before and after the adhesion test. f) Water contact angle of PPMF and FG/PPMF electrode. g) Demonstration of self‐cleaning ability of the FG/PPMF electrode. h) Sheet resistance of the FG/PPMF electrode before and after the self‐cleaning test. i) Relative resistance variation of the FG/PPMF electrode after immersion in water. The inset shows a photograph of the FG/PPMF electrode immersed in water for one week. j) Relative resistance variation of the FG/PPMF electrode after multiple washings test to simulate washing machine environment with water flow shear.

In addition to high conductivity and gas permeability, the FG/PPMF electrode also exhibits mechanical robustness due to the armor design. In this design, gas‐permeable FG fillers are embedded within the PPMF framework, safeguarding them against environmental mechanical forces. First, the FG/PPMF electrode maintains its conductive stability under mechanical bending. As shown in **Figure** **5**a, a square FG/PPMF electrode of 2 cm × 2 cm size exhibits less than a 3% resistance change after bending with tension or compression to different chord lengths. Even after 1000 compression cycles, the resistance change remains below 3%, suggesting the remarkable stability of these sensors during extended usage (Figure 5b). Second, the long‐term wearability of on‐skin electrodes involves continuous friction with the human body or cloth texture. Owing to the armor design, our FG/PPMF electrode proves resilient to such mechanical stresses. A comprehensive evaluation involving 10000 cycles of vertical friction was conducted as shown in Video S4 (Supporting Information). The repeated 10 N mechanical force with a frequency of 4 Hz was applied to strike the FG/PPMF electrode, and the electrical resistance of the electrode was in‐situ monitored. As shown in Figure 5c, during the 10000 cycles of the vertical friction test, the sheet resistance of the FG/PPMF electrode shows almost no change, suggesting the electrode's robust resistance to prolonged mechanical interactions.^[^ ^36^ ^]^ Furthermore, the mechanical robustness of the FG/PPMF electrode is evident in rigorous adhesion tests, where a tape was firmly adhered to the FG/PPMF electrode and then swiftly peeled off to simulate real‐world wear conditions.^[^ ^37^ ^]^ As shown in Figure 5d, there is a negligible impact on the sheet resistance of the electrode after the adhesion test (Figure 5e). This is because the FG filler is securely immersed inside the PPMF framework, remaining unaffected by the tape's adhesive forces (Figure S11, Supporting Information). To elucidate the efficacy of the armor design in safeguarding electrodes against external damage, we conducted a control experiment wherein the same Flash Graphene (FG) ink was deposited onto a polyethylene terephthalate (PET) substrate, without the protective “armor” (Figure S12, Supporting Information). Subsequently, tape adhesion experiments were performed to simulate real‐world wear conditions. Remarkably, the control experiment revealed a substantial change in sheet resistance, indicative of the destruction of a significant portion of the conductive functional materials, which adhered to the tape surface (Figure S13, Supporting Information). This stark contrast between the FG/PPMF electrode and the unprotected FG/PET substrate indicates the pivotal role of the armor design in preserving the integrity and functionality of the electrodes under mechanical force. This control experiment serves as compelling evidence of the protective benefits conferred by the armor design, further validating its importance in enhancing the durability and reliability of flexible electrodes in practical applications.

Additionally, the FG/PPMF electrode demonstrates good water‐resistant performance. The contact angle of the PPMF at room temperature is ≈118.18°, indicative of its inherent hydrophobicity. Upon the incorporation of FG, the contact angle of the FG ink shows a marginal increase to ≈120.42°, affirming the maintenance of hydrophobic properties after FG integration (Figure 5f). The hydrophobic surface imparts a distinctive self‐cleaning attribute to the electrode, where sand and dust particles can be effortlessly washed away by a water jet.^[^ ^25^ ^]^ Here, we utilize calcium carbonate nanoparticles to simulate environmental debris on the FG/PPMF electrode surface. When washed by water flow, the FG/PPMF electrode surface effectively clears white particles, with the continuous water jet reflecting off the sensor surface (Figure 5g and Video S5, Supporting Information). Importantly, the sheet resistance remains unchanged after such rigorous and continuous water jet cleaning, indicating the stability of the FG/PPMF electrode (Figure 5h).

To further demonstrate the waterproof nature of FG/PPMF, repeated immersion tests were conducted.^[^ ^8^ ^]^ By immersing the electrode in water for a period, no discernible trend in sheet resistance change can be observed (Figure 5i), implying the sensor's suitability for extended submersion. The inset displays an optical photograph of the FG/PPMF electrode after one week of immersion, revealing no foreign matter in the water and confirming the enduring adherence of FG to PPMF during prolonged exposure. Combined with the waterproof property and the mechanical robustness of the FG/PPMF electrode, the electrode can be deemed washable. As demonstrated, we immersed the electrode in vigorously stirred water for two hours to simulate the environment in a washing machine and then measured its sheet resistance. After several repeated tests, the sheet resistance remained almost unchanged (Figure 5j and Video S6, Supporting Information). In addition to external forces, environmental changes may also alter the resistance of electrodes, therefore influencing its stability in practical applications. Considering that skin electrodes conform closely to the body's surface, it is crucial to investigate the effects of temperature and humidity on electrode resistance. We confirm that the sheet resistance of FG/PPMF remains consistent across different temperature and humidity conditions (Figure S14a, Supporting Information). Additionally, direct contact with sweat on the body surface may induce signal instability. Hence, we further verified the saline‐resistant property of FG/PPMF (Figure S14b, Supporting Information). Based on these findings, we believe that the characteristics of FG/PPMF are well‐suited for potential applications as on‐skin electrodes. A comparative table contrasting our FG/PPMF composite with previously reported on‐skin electronics is presented in Table S1 (Supporting Information). The table suggests that our composite offers improvements over existing technologies, including enhanced gas permeability, superior mechanical robustness, comparable high electrical conductivity and potentially lower cost. Moreover, the cost per FG/PPMF electrode (2 cm × 2 cm) is only 1.39 cents after accounting for major factors (Note S1, Supporting Information), which is priced at one‐third the cost of commercial Ag/AgCl gel electrodes.

**Figure 6:**
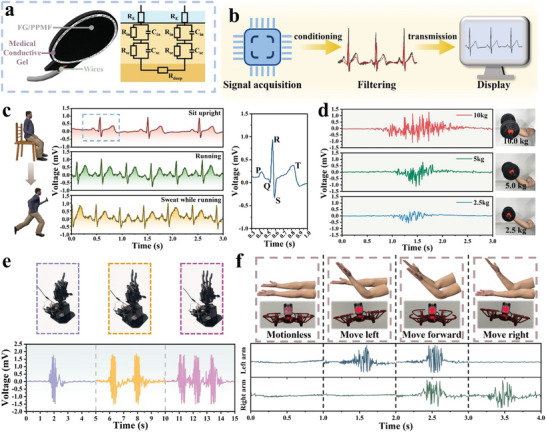
ECG and EMG measurements by using FG/PPMF electrode patch. a) The schematic illustration of the FG/PPMF electrode patch and the equivalent circuit model when monitoring electrophysiology signals. b) Schematic depiction of the processing of the physiological electrical signals collected by the FG/PPMF electrode patch. c) ECG Recordings using FG/PPMF electrode patches from volunteers in different states: sitting upright, running, and sweating while running. The right subgraph displays the standard characteristic peaks. d) EMG recordings using the FG/PPMF electrode patch as volunteers lift dumbbells weighing 2.5 kg, 5 kg, and 10 kg, respectively. e) Real‐time control of a robotic arm using EMG signals collected by the FG/PPMF electrode patch. f) Modulating the motion state of the UAV through EMG signals.

Due to its lightweight nature, high conductivity, flexibility, gas permeability, and mechanical robustness, the FG/PPMF electrode proves to be an ideal candidate for on‐skin electronics dedicated to monitoring human physiological electrical signals.^[^ ^38^ ^]^ To achieve precise signal collection and comprehensive human health monitoring, the FG/PPMF electrode was first assembled into a three‐lead electrode patch,^[^ ^39^ ^]^ as detailed in Figure S15 (Supporting Information). Then the FG/PPMF electrode patch was attached tightly to the volunteer's skin using medical adhesive tape. The equivalent circuit diagram of the FG/PPMF electrode patch on the skin surface is depicted in **Figure** **6**a.^[^ ^40^ ^]^ During the test, our FG/PPMF electrode patch exhibited lower contact impedance compared to the commercial Ag/AgCl gel electrode patch (Figure S16, Supporting Information). This characteristic enables the FG/PPMF electrode to sensitively capture weak muscle electrical signals and physiological electrical signals. After being processed by a chip, transmitted to a PC via Bluetooth, and further processed by a digital filter on the PC's Arduino IDE platform, the captured weak physiological electrical signal was ultimately transformed into clear physiological signal waveforms (Figure 6b).

When applied to a volunteer's chest and upper arm (Figure S17, Supporting Information), the FG/PPMF electrode patch exhibits the capability to record high‐quality electrocardiogram (ECG) signals and electromyogram (EMG) signals, respectively. The quality of the ECG signals obtained was even slightly better than the commercial Ag/AgCl gel electrode patch (Figure S18a, Supporting Information). Monitoring ECG signals in different states demonstrates the stable monitoring capability of the FG/PPMF electrode patch. In a sitting still state, the volunteer's ECG signals display stable and periodic cardiac waves, with a magnified segment showcasing the P‐wave, QRS complex, and T‐wave (Figure 6c).^[^ ^41^ ^]^ Compared to a commercial Ag/AgCl gel electrode patch, our FG/PPMF electrode patch exhibits a higher signal‐to‐noise ratio (SNR) of 38.7 dB, resulting in a clearer representation of cardiac features, particularly beneficial in highlighting atrial fibrillation characteristics.^[^ ^42^ ^]^ When the volunteer transitions from standing to jogging, ECG signals exhibit notable changes, including increased signal amplitude and heart rate, indicative of intensified cardiac activity. It should be noted that motion artifacts and friction between electrodes and skin normally contribute to elevated noise levels during jogging. And sweat further impacts signal impedance and introduces additional noise. While our FG/PPMF electrode patch exhibits gas permeability and superior thermal conductivity (Figure S19, Supporting Information), which not only prevents sweat accumulation but also benefits heat dissipation, thus effectively controlling sweat during running tests and improving thermal comfort during exercise wear. As a result, valuable ECG signals can still be obtained during running with sweating, maintaining a reference‐worthy signal with an SNR of 22.1 dB.

When attached to the arm, the FG/PPMF electrode patch effectively detects electromyographic signals from various muscle contraction states. As shown in Figure 6d, the volunteer's electromyogram (EMG) signal remains stable at 0 mV with a small magnitude in a relaxed state. When the volunteer bends the elbow with a 2.5 kg dumbbell, the muscle contracts and generates weak physiological electrical signals. As a result, the EMG signal exhibits short‐time fluctuations (≈750 ms) with a mid‐range magnitude of 0.36 mV. When increasing the dumbbell weight to 5 kg and 10 kg, physiological electrical signals will be further heightened, leading to higher EMG signals of 0.77 mV and 1.09 mV, respectively. During EMG monitoring, the FG/PPMF electrode patch also demonstrates a high SNR of 34.5 dB, which is comparable to a commercial Ag/AgCl gel electrode patch (Figure S18b, Supporting Information). Furthermore, the collected EMG signals could be utilized in human‐machine interfaces, enabling the control of a robotic hand to assist individuals with hand disabilities. The sensing system, which is connected to the robotic hand via BLE 4.0, can effectively translate muscle electromyogram peaks into predefined commands, allowing the robotic hand to perform gestures corresponding to the detected muscle activity, such as “1”, “2” and “3” gestures, as demonstrated in Figure 6e and Video S7 (Supporting Information). The FG/PPMF electrode not only supports three electrodes for single‐channel control of a robotic hand to gesture representing numbers but is also suitable for a multi‐channel human‐machine interface for controlling Unmanned Aerial Vehicle (UAV). In Figure 6f and Video S8 (Supporting Information), when the volunteer's upper arm is relaxed, the UAV hovers. When the left upper arm contracts, the UAV moves left. Simultaneous contraction of both upper arms makes the UAV fly forward. Contraction of the right upper arm initiates a rightward flight.

**Figure 7:**
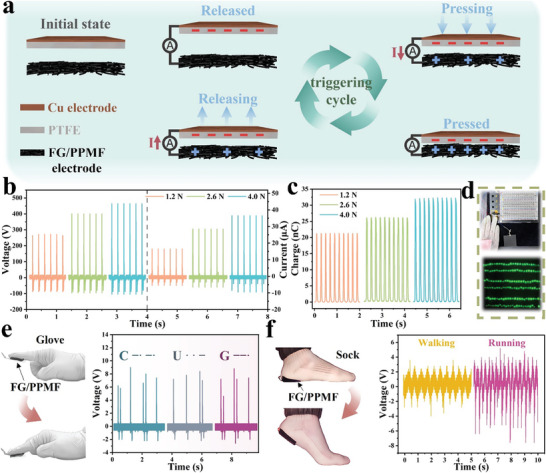
Demonstration of FG/PPMF electrode as triboelectric nanogenerator (TENG) sensor. a) Schematic representation of the FG/PPMF TENG in double‐electrode mode. b,c) Open‐circuit voltage and short‐circuit current (b), and transferred charge amount (c) of the FG/PPMF TENG in double‐electrode mode under various pressure conditions. d) Optical images showing energy harvested in double‐electrode mode FG/PPMF TENG, capable of illuminating up to 150 LEDs. e) Integration of the FG/PPMF TENG into a glove, tapping on PET to convey the “CUG” message in Morse code. f) Integration of the FG/PPMF TENG into a sock for monitoring different walking conditions.

Finally, the high flexibility, mechanical robustness, and excellent triboelectric output of the FG/PPMF electrode also enable its suitability for TENG based self‐powered sensors.^[^ ^43^ ^]^ Moreover, the FG/PPMF electrode can function as both the electrode and tribomaterial (**Figure** **7**a), eliminating the need for additional electrodes to collect and transmit current generated by friction.^[^ ^44^ ^]^ To evaluate the electrical performance of the FG/PPMF based TENG, polytetrafluoroethylene (PTFE) was utilized as the tribo‐negative electrode. Surface containing FG in contact with PTFE during testing. By applying impact forces (at a frequency of 5 Hz) ranging from 1.2 N to 4.0 N to the TENG sensor with a 2 cm × 2 cm friction area, a scalable electric power generation capability was demonstrated. As shown in Figure 7b,c, as the force increases, the open circuit voltage (V_oc_), short circuit current (I_sc_), and charge density increase, reaching impressive values of 464 V, 36.5 µA, and 32.3 nC, respectively, at 4.0 N. In addition to the magnitude of the impact force, the frequency of impact also affects the triboelectric performance of the TENG. As the impact frequency increases, the initial V_oc_ and I_sc_ gradually increase, reaching their maximum values at a frequency of 7 Hz, with V_oc_ at 504 V, I_sc_ at 37.5 µA, and a charge transfer of 34.5 nC (Figure S20, Supporting Information). Notably, the corresponding V_oc_ values are 82 V and 49.6 V when using traditional copper as electrodes or when using the PPMF surface of FG/PPMF composite material in contact with PTFE to form a TENG for power generation (Figure S21, Supporting Information). The enhanced electrical output of FG/PPMF in the TENG can be attributed to the uniform distribution of FG ink within the pores of PPMF, which provides a larger effective contact surface area. Moreover, due to the nanoporous structure of the FG assembly, charge transport and storage are facilitated for high triboelectric output. Harnessing this high‐performance triboelectric effect of FG/PPMF TENG sensor, we successfully illuminate 150 LEDs using the harvested mechanical energy (Figure 7d and Video S9, Supporting Information), which demonstrates the potential of FG/PPMF as a portable power source.^[^ ^45^, ^46^, ^47^ ^]^

The remarkable efficiency in power generation exhibited by the FG/PPMF TENG allows its use as a TENG based wearable pressure sensor, which could be integrated into garments for human motion detection. In Figure S22a (Supporting Information), we illustrate the direct integration of FG/PPMF into a glove, functioning as a single‐electrode mode TENG capable of sensitively detecting finger tap motions. As depicted in Figure 7e and Video S10 (Supporting Information), participants wearing a glove with integrated FG/PPMF sensors executed Morse code tapping on a PET surface (one tap for a dot signal “·”, two successive taps for a long signal “‐”). Each tap generated distinct and well‐defined peaks, enabling precise identification of the transmitted “CUG” message. Expanding its application beyond tap motion detection, we integrated the gas‐permeable TENG sensor (6 cm × 6 cm) into a sock to monitor human walking status (Figure S22b and Video S11, Supporting Information). The TENG sensor generates electric signals when the foot separates from the shoe, and the signal changes in sync with the walking speed. Figure 7f depicts the electrical signals under different walking conditions, such as walking and running, revealing distinct peaks corresponding to each lift and step during walking. The transition to running introduces higher friction, resulting in an increased frequency and larger peak in the electrical signal. All these results demonstrate the exceptional gas permeability, flexibility, conductivity, and high triboelectric properties of FG/PPMF, positioning it as a promising self‐powered wearable sensor for real‐time monitoring of human body movements.

## 3 Conclusion

In summary, we have demonstrated a mechanically robust and gas‐permeable on‐skin FG/PPMF electrode by integrating the “armor” design strategy and the turbostratic feature of FG. We devised a straightforward method by embedding highly gas‐permeable FG assembly fillers within the protective porous PPMF substrate. Synthesized through the non‐equilibrium FJH process, FG's turbostratic characteristic prevents undesirable AB‐stacking, ensuring a continuous conductive network with nanopore structures. This assembly, coupled with a customized screen‐printing technique, resulted in a robust, gas‐permeable multifunctional on‐skin electrode. With a low sheet resistance of 125.2 ± 4.7 Ω/□ and exceptional gas permeability (≈10.08 mg cm^−2^ h^−1^), the FG/PPMF electrode exhibited remarkable stability, surviving adhesion and washable tests, and enduring 10000 cycles of mechanical contact. When used as sensors, FG/PPMF electrodes excelled in physiological signal monitoring, human‐machine interaction, and triboelectric energy generation. With these advantages, our research contributes to the potential applications of multifunctional integrated flexible wearable sensors, such as health monitoring, behavioral monitoring, and human‐computer interaction. It holds great promise for future scalable, high‐performance flexible sensing applications.

## 4 Experimental Section

### Materials

Carbon black was offered by Tianjin Huayuan Chemical Technology Co., Ltd. DBE was supplied by Shandong Ruigang Chemical Co., Ltd. VC‐Vac resin (CP‐710) was acquired by Guangzhou Rongcheng Chemical Co., Ltd. Polypropylene melt‐blown nonwoven fabrics (25 g m^−2^) were purchased from Alibaba Group Holding Ltd. All chemicals were purchased as was without further purification.

### Synthesis of FG

Flash Joule Heat (FJH) purchased from Shenzhen ZhongKeJingYan Technology Co., Ltd. In summary, 120 mg of Carbon black was placed within a quartz tube (6 mm internal diameter), and a tension spring was wrapped around the tube to maintain compression on the Carbon black inside. Graphite cubes were positioned alongside copper wool on either side of the Carbon black. The slightly smaller size of the graphite cubes allowed for the expulsion of exhaust gas during the FJH process, while the copper wool served to prevent direct contact between the electrodes and the carbon source. The FJH process necessitates the evacuation of the reaction vessel beforehand, enabling gas removal from the quartz tube throughout the FJH procedure. 10x of 430 V, 10 mF capacitors, were employed for discharge. The discharge voltage was set at 220 V, and the discharge duration was precisely controlled within 500 ms. Post the FJH process, the FG can be directly pushed out from the quartz tube.

### Preparation of FG Ink

Finely ground FG of 2 g was sieved through a mesh with a screen size of 150 µm and subsequently incorporated into 0.38 g VC‐Vac resin dissolved in 5 mL DBE solution. The mixture was milled by ball milling at a speed of 500 r min^−1^ for 11 hours. After separation from the milling balls, the resulting black thick ink could be directly utilized for screen‐printing. The solid content of the prepared ink was ≈25.5%.

### Preparation of the FG/PPMF Electrodes via the Screen Printing Technique

The FG/PPMF electrodes were fabricated using the commercially scalable screen‐printing method. Initially, screen meshes with different patterns were designed. Subsequently, polypropylene melt‐blown nonwoven fabrics were positioned beneath the screen mesh, and both were securely fixed to the printer. Once installed, the FG ink was applied to the screen mesh, and the patterns were printed onto the fabrics by moving the squeegee over a stencil at an appropriate speed. The resulting electrodes, labeled FG/PPMF, were allowed to naturally dry for 1 hour at 60 °C.

### FG/PPMF as ECG and EMG Electrode

The detailed electrode preparation was illustrated in Figure S15 (Supporting Information). Prior to attachment to the body, all electrodes underwent thorough cleaning with deionized water. A small amount of medical conductive gel (≈0.25 mL) was applied to enhance the contact between the electrodes and the skin. The electrode placement positions for ECG and EMG tests were outlined in Figure S17 (Supporting Information). All tests were conducted at room temperature.

### FG/PPMF as TENG Based Self‐Powered Sensor

The FG/PPMF can be utilized directly as a TENG based self‐powered sensors without requiring additional processing.

### FG/PPMF Biocompatibility Test

FG/PPMF underwent washing and UV irradiation for 30 minutes. It was then soaked in DMEM for 24 hours to prepare the extraction solution. The L929 cell suspension was subsequently transferred to a 96‐well plate and incubated for 24 hours. Following this, the culture medium was removed and replaced with FG/PPMF extraction solution, and the plate was further incubated at 37 °C for an additional 24 hours. Following this incubation period, live and dead cells were stained with diluted dyes of Calcein‐AM and PI, respectively, and observed under a fluorescence microscope (ZEISS, AxioVert.A1).

### Characterization and Measurements

Materials characterization was conducted by field emission scanning electron microscope (FESEM, Hitachi SU8010), Transmission electron microscope (TEM, JEOL JEM‐F200), X‐ray diffraction (XRD, Bruker D8 advance X‐ray diffractometer), Raman spectroscopy (Renishaw inVia, 532 nm), X‐ray photoelectron spectroscopy (XPS, Thermo Scientific K‐Alpha). Electrical conductivity was tested by a 4‐point probe resistivity measurement system (RTS‐4). The open‐circuit voltage of the TENG was measured using a digital phosphor oscilloscope (MSO 2024B, Tektronix, Inc., Beaverton, OR, USA) and the electrical measurements were measured using a low‐noise current preamplifier (model no. SR570, Stanford Research Systems, Inc., Sunnyvale, CA). The charge transfer from the output signals was measured using an electrometer (Keithley 6514, Cleveland, OH, USA).

In the vertical friction experiments, vertical friction was applied on the FG/PPMF surfaces using a vibration exciter. Acrylic sheets, a commonly used material, were employed as the friction interface to assess the durability of the FG/PPMF, where the friction area was 2 cm × 2 cm and the friction force was 10 N, the sheet resistance of the sample was recorded after every 1000 friction cycles. Furthermore, in the tape adhesion experiment, common adhesive tape was firmly adhered to the surface of FG/PPMF for 5 minutes, then slowly peeled it off. The sheet resistance of FG/PPMF was subsequently tested before and after the tape adhesion experiment, respectively. Gas permeability tests were conducted in a constant temperature and humidity chamber throughout the entire experiment. The chamber's temperature was maintained at 35 °C, with humidity controlled within the range of 45–55% RH. Glass bottles, each filled with 5 ml of deionized (DI) water, were sealed with various samples. The real‐time weights of the bottles were recorded at 24‐hour intervals, and the observed changes were utilized to calculate the average water loss over the five‐day duration. The repeated immersion tests were conducted with a soaking period of 1 hour, followed by drying at 60 °C to measure the sheet resistance. In the washing machine environmental simulation tests, a 2‐hour cleaning cycle was employed, followed by drying at 60 °C to measure the sheet resistance.

### Ethical Information for Studies Involving Human Subjects

The experiments involving human subjects strictly adhered to the guidelines established by the Institutional Review Board and received thorough scrutiny and approval from the Ethics Committee of Nanjing Agricultural University (Approval Number: NJAU.No20220622135). All participants in the studies volunteered willingly and provided informed consent. As the electrodes were placed on the users' bodies, a restricted amount of necessary identifiable images was utilized. Importantly, all identifiable information used was explicitly consented to by the participants.

## Conflict of Interest

The authors declare no conflict of interest.

## Supporting information

Supporting Information

Supplementary Video S1

Supplementary Video S2

Supplementary Video S3

Supplementary Video S4

Supplementary Video S5

Supplementary Video S6

Supplementary Video S7

Supplementary Video S8

Supplementary Video S9

Supplementary Video S10

Supplementary Video S11

## Data Availability

The data that support the findings of this study are available from the corresponding author upon reasonable request.
