# Supplementary material for: Bioinspired Robust Gas‐Permeable On‐Skin Electronics: Armor‐Designed Nanoporous Flash Graphene Assembly Enhancing Mechanical Resilience
Source: Adv Sci (Weinh). 2024 May 5;11(26):2402759. doi: 10.1002/advs.202402759 (PMC11234450; doi:10.1002/advs.202402759)
Supplement: Supplementary file 1 — Supporting Information [file ADVS-11-2402759-s004.pdf]

ADVANCED SCIENCE
Open Access

Supporting Information

for *Adv. Sci.*, DOI 10.1002/advs.202402759

Bioinspired Robust Gas-Permeable On-Skin Electronics: Armor-Designed Nanoporous Flash Graphene Assembly Enhancing Mechanical Resilience

Yang Chen, Zixuan Liu, Zhigang Wang, Ying Yi, Chunjie Yan, Wenxia Xu, Feng Zhou, Yuting Gao, Qitao Zhou, Cheng Zhang* and Heng Deng**

Supporting Information for

Bioinspired Robust Gas-Permeable On-Skin Electronics: Armor-Designed Nanoporous Flash Graphene Assembly Enhancing Mechanical Resilience

Yang Chen [a, #], Zixuan Liu [b, #], Zhigang Wang [a], Ying Yi [c], Chunjie Yan [a], Wenxia Xu [a], Feng Zhou [a], Yuting Gao [a], Qitao Zhou [a,] *, Cheng Zhang [b,] *, and Heng Deng [a, d,] *

[a] *Faculty of Materials Science and Chemistry, China University of Geosciences, Wuhan, 430074, People's Republic of China*

[b] *College of Engineering, Nanjing Agricultural University, Nanjing, 210031, People's Republic of China*

[c] *School of Mechanical Engineering and Electronic Information, China University of Geosciences, Wuhan, 430074, People's Republic of China*

[d] *Shenzhen Research Institute, China University of Geosciences, Shenzhen, 518000, People's Republic of China*

Email: zhouqitao@cug.edu.cn; zhangcheng@njau.edu.cn; dengheng@cug.edu.cn.

# These authors contribute equally to this work

Supplemental Figures

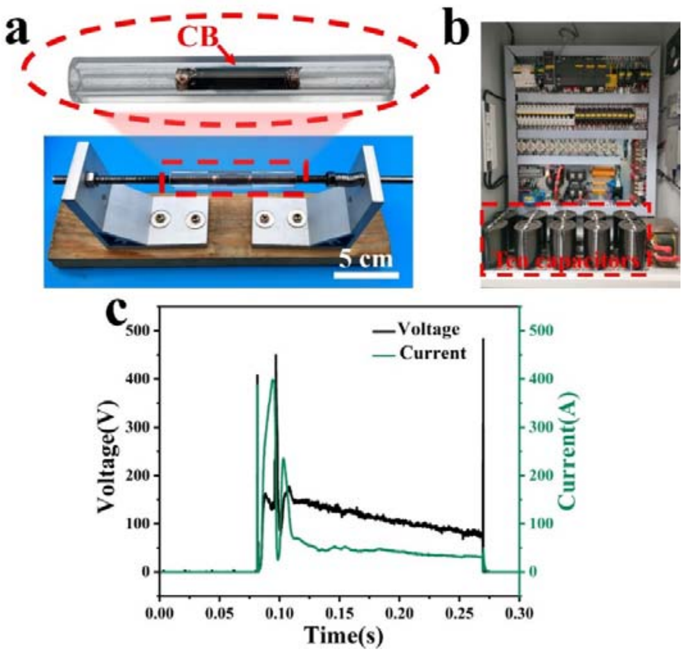

Figure S1. a) Optical image of the reaction vessel with the CB. b) Ten parallel capacitors supply the FJH process. c) Voltage and current flow through CB during FJH, with the reaction concluding within milliseconds.

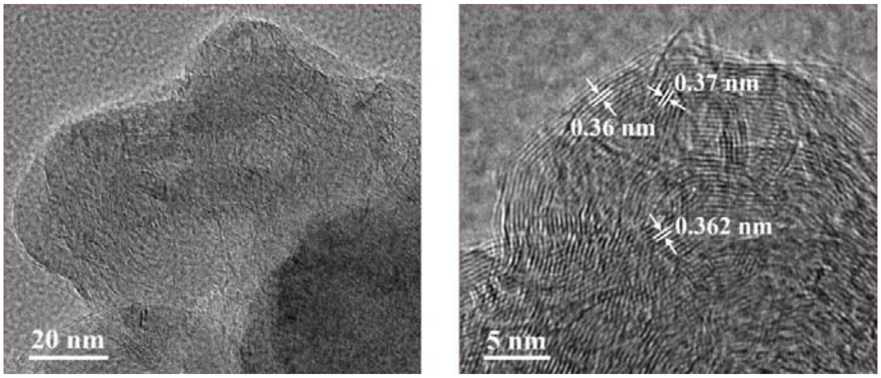

Figure S2. HR-TEM image of CB particle in different magnifications.

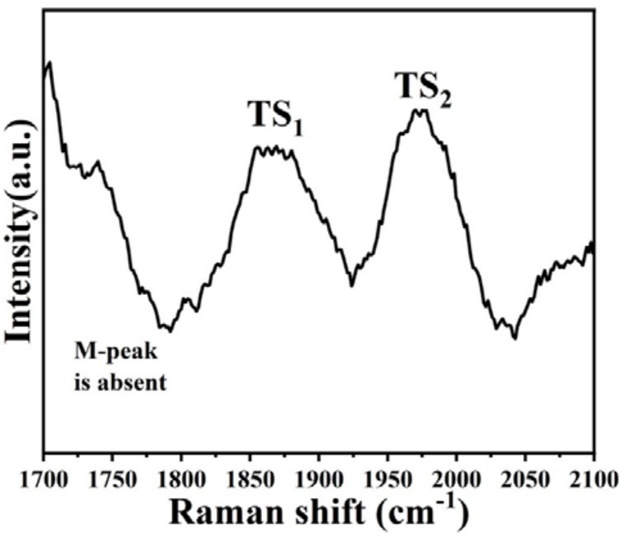

Figure S3. Raman spectrum of FG focusing on the range between 1700 cm^{-1} and 2100 cm^{-1} to show the TS_1, TS_2 peaks and absent M-peak within this range.

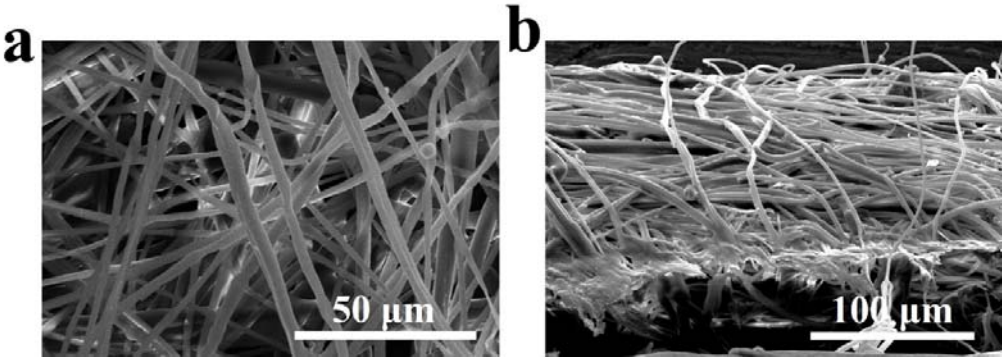

Figure S4. a) SEM image of PPMF. b) Cross-sectional SEM image of PPMF.

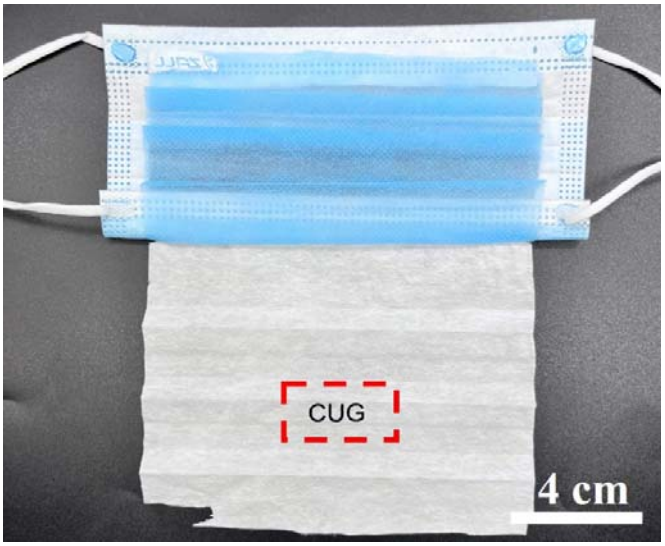

Figure S5. The PPMF layer on the medical mask can be disassembled and directly printed with FG ink, yielding a printing effect that is indistinguishable from the experimental PPMF.

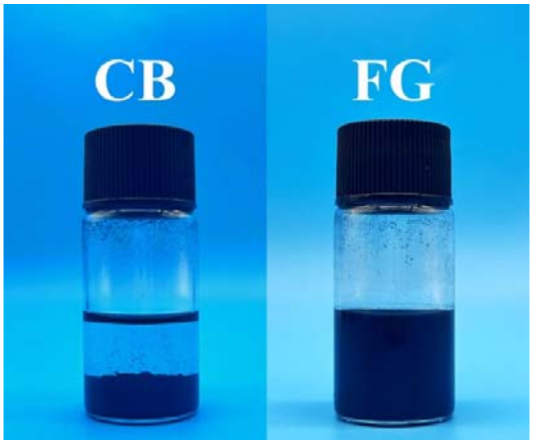

Figure S6. CB and FG dispersion tests in DBE. CB and FG were homogeneously dispersed in DBE at 2 mg/mL by sonication. After a day of settling, the CB naturally settled in the DBE, while the FG remained uniformly dispersed in the solution.

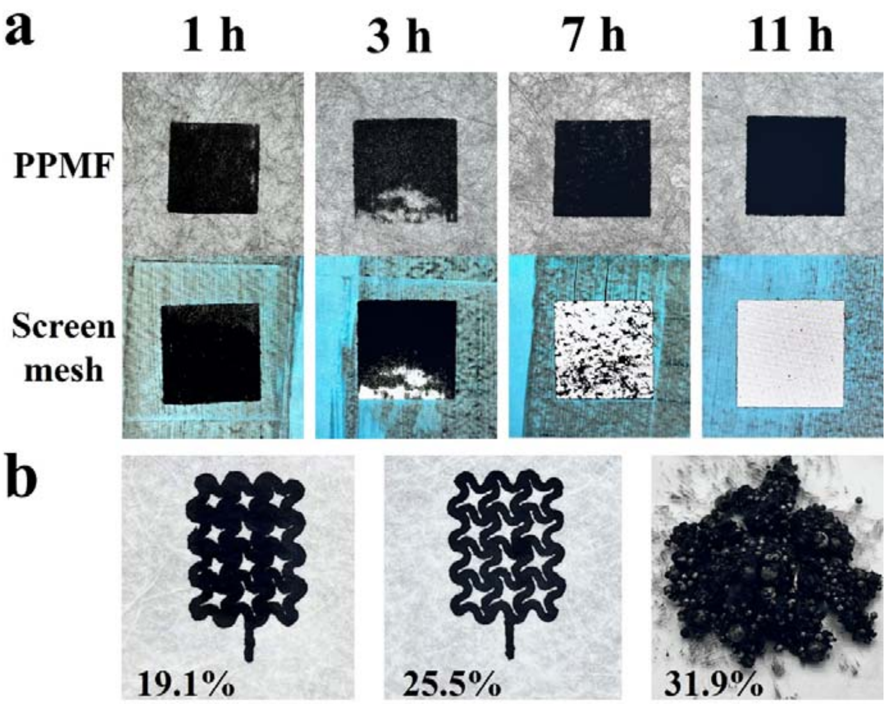

Figure S7. Factors affecting FG inks. a) Images of inks with different ball milling times on PPMF and screen mesh after printing. b) Effect of different solid contents on inks.

Note: Printing was conducted utilizing inks derived from varying ball milling durations (Figure S7a). Examination of ink loadings on nonwovens and screen meshes indicates that the functional material particles within the ink diminish in size and enhance mobility with prolonged ball milling durations. Sufficient ball milling time proves advantageous for FG ink printing. Inks comprising 19.1%, 25.5%, and 31.9% solid content were formulated and employed for printing post an 11-hour ball milling process (Figure S7b). The ink with 19.1% solid content exhibits extensive spreading, whereas the 31.9% solid content ink fails to form a paste. Furthermore, inks with 31.9% solids pose challenges in separation due to FG agglomeration and its adhesion to the balls post-adequate ball milling. Conversely, the ink with 25.5% solid content achieves flawless printing, effectively showcasing customized patterns.

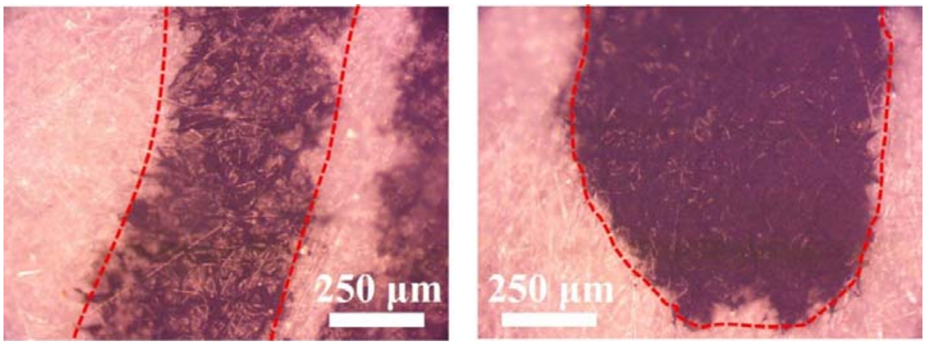

Figure S8. Microscope image of the electrode pattern printed by FG ink. The resolution of printing can be as low as 500 μm.

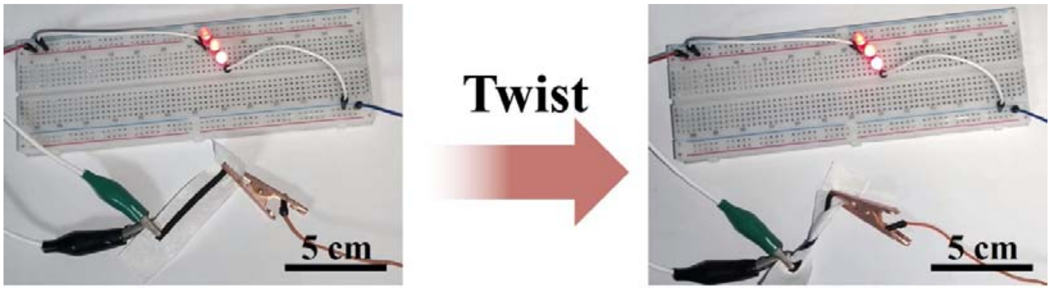

Figure S9. The conductive pathways created with FG inks retain robust pathways even under extreme distortion conditions.

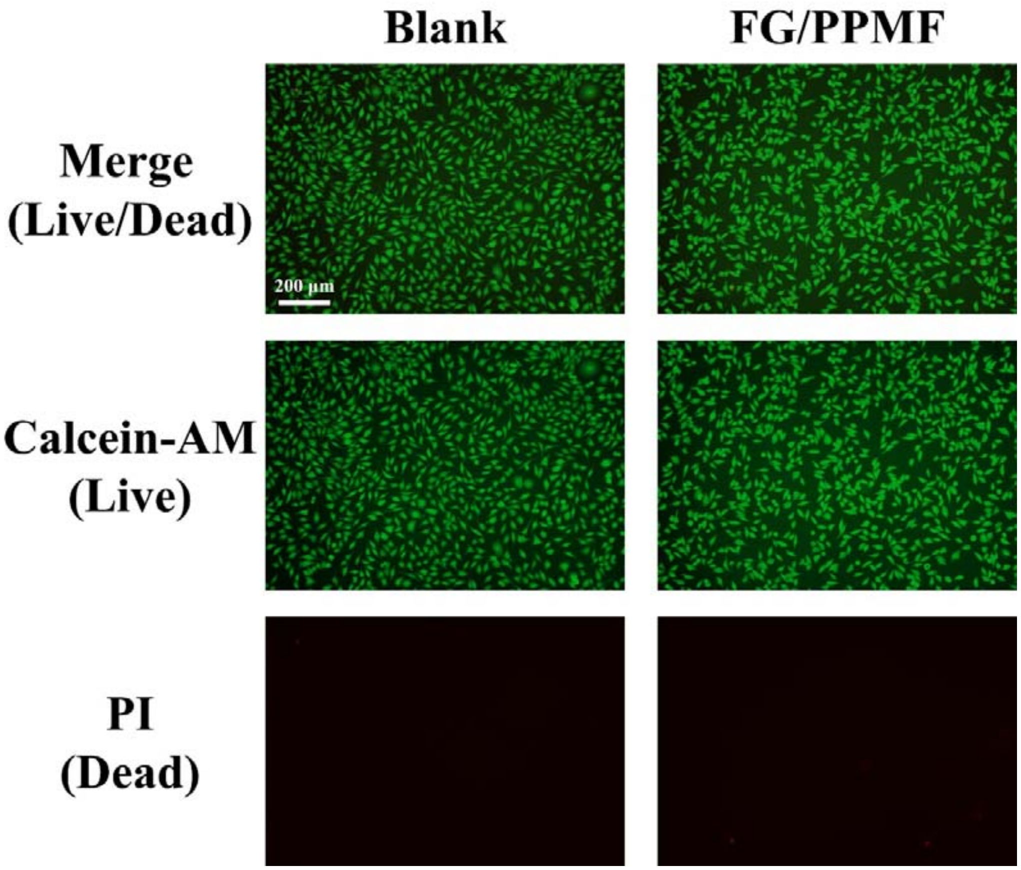

Figure S10. Fluorescence microscopy images of L929 cells cultured for 24 h. L929 cells co-cultured with FG/PPMF exhibited morphologically normal characteristics and showed no significant signs of cell death, an image similar to that observed in the control group.

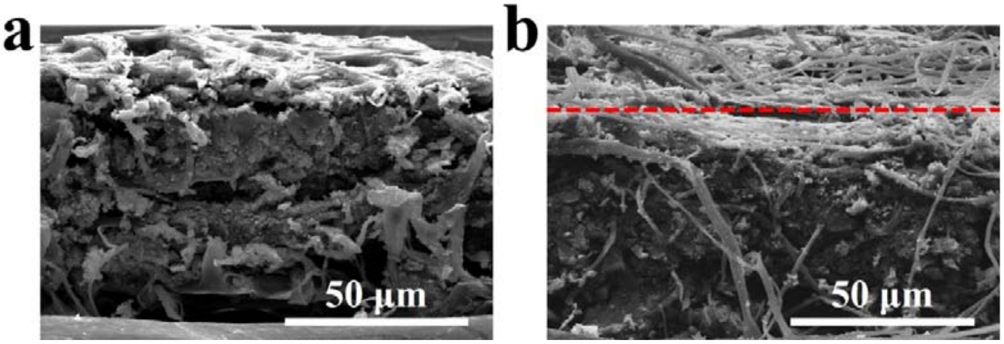

Figure S11. Cross-sectional SEM images of FG/PPMF a) before adhesion test, b) after the adhesion test.

Note: PPMF serves as armor safeguarding the conductive path formed by the internal functional materials from powerful external adhesive forces. Post adhesion tests, only the fibers in the surface layer of FG/PPMF were impacted, while cross-sectional SEM analysis affirmed the preservation of the functional materials within the fibers.

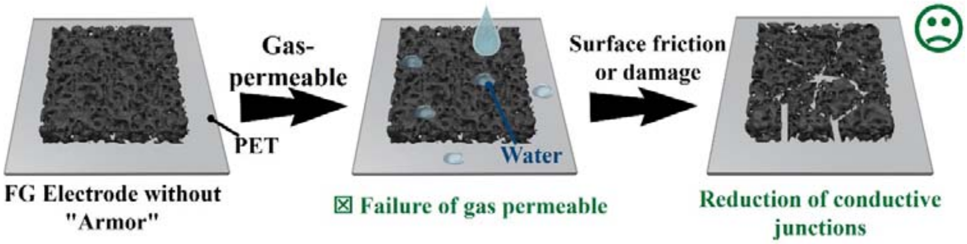

Figure S12. Schematic representation depicting the performance of an electrode without "armor design," created by printing FG ink onto PET. The non-porous substrate struggles to offer gas-permeable channels, and the direct exposure of conductive functional materials without "armor" protection is susceptible to damage from external forces.

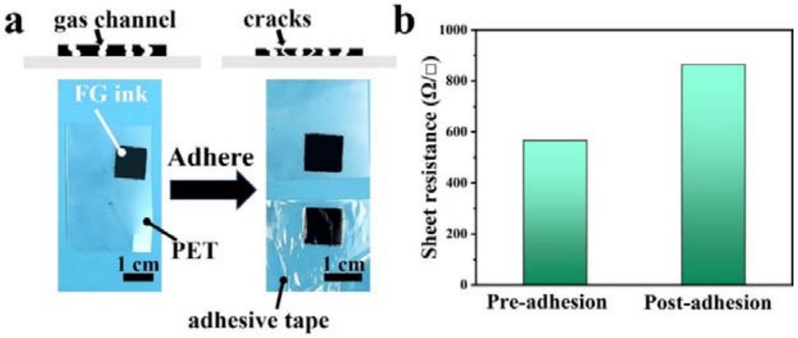

Figure S13. a) Tape adhesion experiment by depositing FG on PET substrate. Without the "armor design", the tape can easily adhere to a large amount of FG and destroy the conductive junctions. b) Sheet resistance of electrode without "armor design" before and after adhesion of tape.

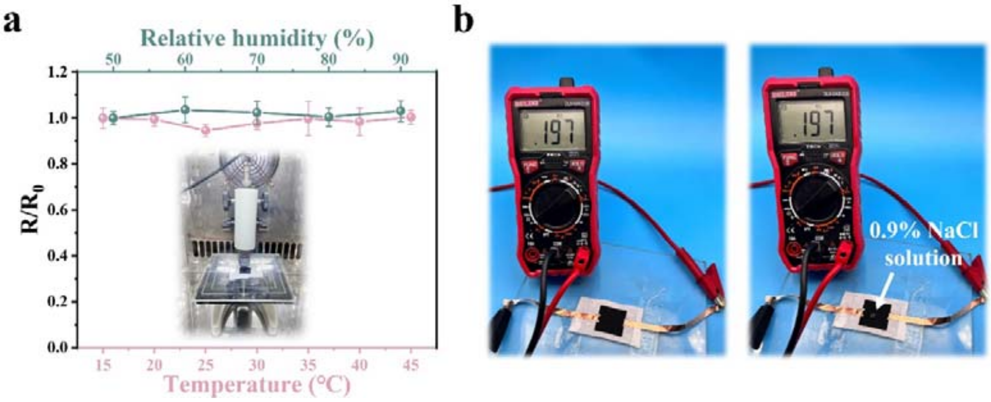

Figure S14. a) The variation in the sheet resistance of the FG/PPMF electrode was examined under different temperature and humidity conditions. b) The resistance of the FG/PPMF electrode remained stable before and after the addition of 0.9% NaCl solution.

Note: The test of FG/PPMF in Figure S14a was conducted by placing the RTS-4 in a constant temperature and humidity chamber, as illustrated in the inset. Temperature

effects were tested at 50% relative humidity, while humidity effects were assessed at a temperature of 25°C.

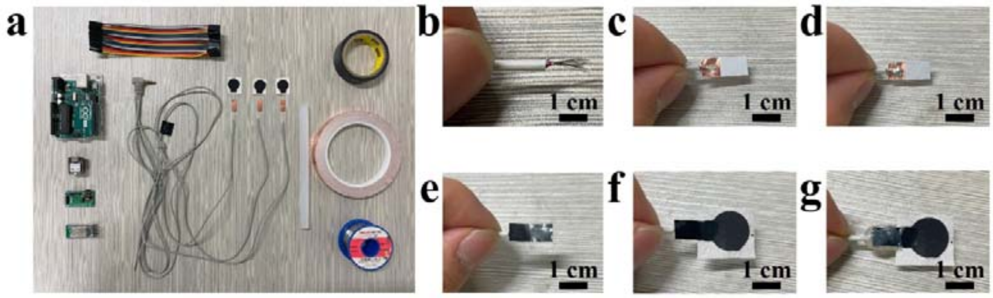

Figure S15. a) Electronic components and materials required for physiologic electrical monitoring. b-g) the three-lead FG/PPMF electrode patch preparation process.

Note: To achieve wearable physiological signal monitoring, the required electronic components include the Arduino Uno development board, Seeed Xiao RP2040 microcontroller unit (MCU), Kingsense company's KS1081 test board, Shenzhen DaXia Longque's BT-24 Bluetooth module, and DuPont wires.[1] To prepare the three-lead FG/PPMF electrode patch, the necessary materials include three FG/PPMF, a three-lead cable with shielding and a 3.5 mm Tip-Ring-Sleeve (TRS) connector, conductive copper tape, solder, insulation tape, and hot melt glue. The first step is to separate one end of the three-lead cable, and trim off the insulation and shielding, leaving approximately 5 mm of exposed signal wire (Figure S15b). The second step is to cut a piece of conductive copper tape measuring about 5 mm × 15 mm, peel off the release paper from around 5 mm of the tape, and affix the exposed signal wire to the copper tape (Figure S15c). The third step is to use a soldering iron to melt the solder

and evenly spread it at the junction of the signal wire and the copper tape (Figure S15d). The fourth step is to cut a piece of insulating tape measuring about 5 mm × 15 mm and tightly adhere it to the back of the junction between the signal wire and the copper tape, protecting the copper tape from breaking and preventing interference from direct skin contact (Figure S15e). The fifth step is to peel off the remaining release paper from the copper tape affix the copper tape to the pins of the FG/PPMF electrode patch, and apply appropriate pressure to secure it (Figure S15f); the last step is to use hot melt glue to encapsulate the junction of the cable/conductive copper tape and the junction of the conductive copper tape/ FG/PPMF electrode patch, enhancing the connection strength while shielding against external interference (Figure S15g).

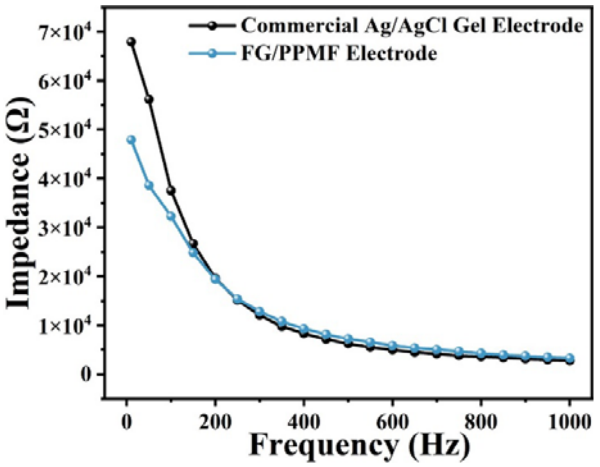

Figure S16. Interface contact impedance between the skin and different electrodes.

Note: The results indicate that in the low-frequency range (0-200 Hz), the contact impedance between FG/PPMF electrodes and the human body is superior to commercial Ag/AgCl gel electrodes. In the high-frequency range (200-1000 Hz), the contact impedance between FG/PPMF electrodes and the human body is comparable to

commercial Ag/AgCl gel electrodes. Considering that ECG and EMG signals typically fall within the low-frequency range (ECG: <27.5 Hz; EMG: <37 Hz),[1-2] the contact impedance between FG/PPMF electrodes and the human body is lower in this frequency range. Consequently, FG/PPMF electrodes can more sensitively capture faint ECG and EMG signals.

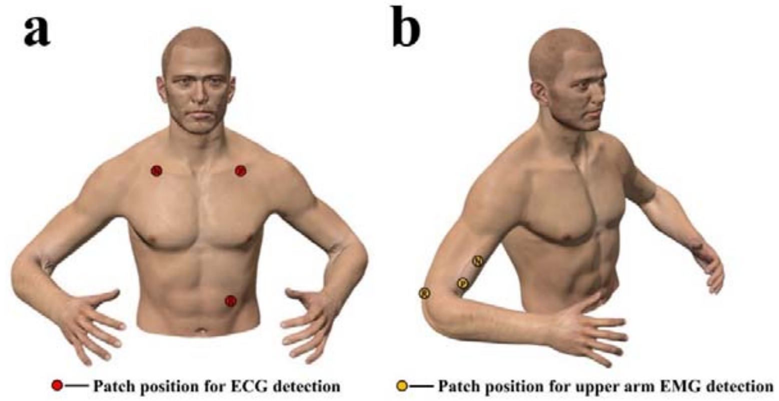

Figure S17. The three-lead FG/PPMF electrode patch paste position when monitoring a) ECG, b) upper arm EMG.

Note: During ECG monitoring, the reference electrode is placed along the mid-clavicular line at the sixth intercostal space, while the positive electrode and negative electrode are affixed at the first intercostal space along the mid-clavicular line on the left and right sides, respectively. During upper arm EMG monitoring, the reference electrode is placed at the junction of the medial head of the triceps brachii and the olecranon, the positive electrode is affixed to the anterior end of the long head of the biceps brachii, and the negative electrode is placed at the posterior end of the long head of the biceps brachii.

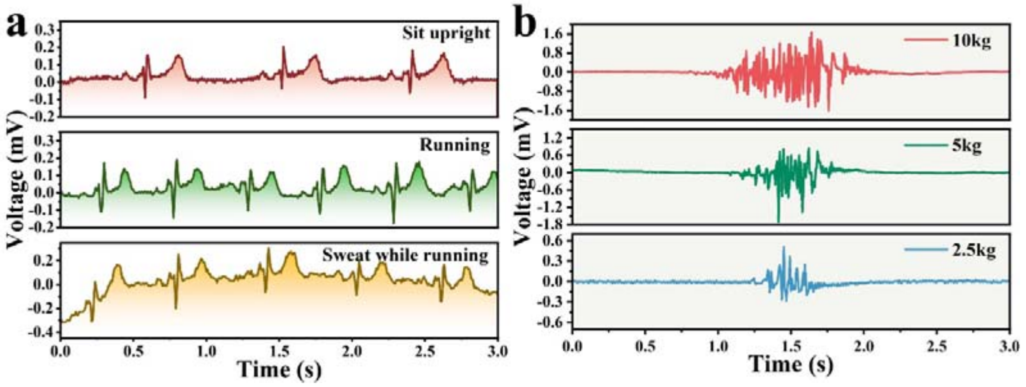

Figure S18. a) ECG signals monitored by commercial Ag/AgCl gel electrode patches in different body states. b) EMG signals monitored by commercial Ag/AgCl gel electrode patches in different muscle contraction states.

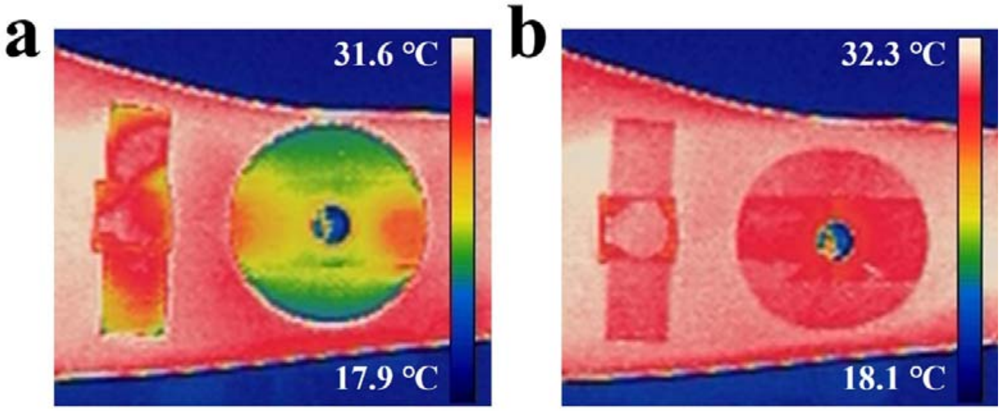

Figure S19. Surface thermographic images of commercial Ag/AgCl gel electrode patches and FG/PPMF electrode patches a) immediately after application to the forearm, b) after 5 min of application to the forearm.

Note: Heat dissipation capability is a crucial indicator in electrode comfort management.[3] While volunteers are in a seated position, both commercial Ag/AgCl gel electrode patches and FG/PPMF electrode patches are applied to the left forearm. Immediately after application, thermal images of the central regions of commercial

Ag/AgCl gel electrode patches and FG/PPMF electrode patches are recorded (Figure S19a). It can be observed that the central region temperature of FG/PPMF electrode patches is significantly higher than that of commercial Ag/AgCl gel electrode patches. This is attributed to the excellent thermal conductivity of graphene. Compared to commercial Ag/AgCl gel electrode patches, FG/PPMF electrode patches can quickly adapt to the body temperature, and relieves stuffiness that occurs during prolonged wear. After both commercial Ag/AgCl gel electrode patches and FG/PPMF electrode patches have been on the volunteer's forearm for 5 min, their surface temperatures become quite similar (Figure S19b). It means that the commercial Ag/AgCl gel electrode patch has a hysteresis on body temperature conduction, which is not beneficial for heat dissipation during exercise.

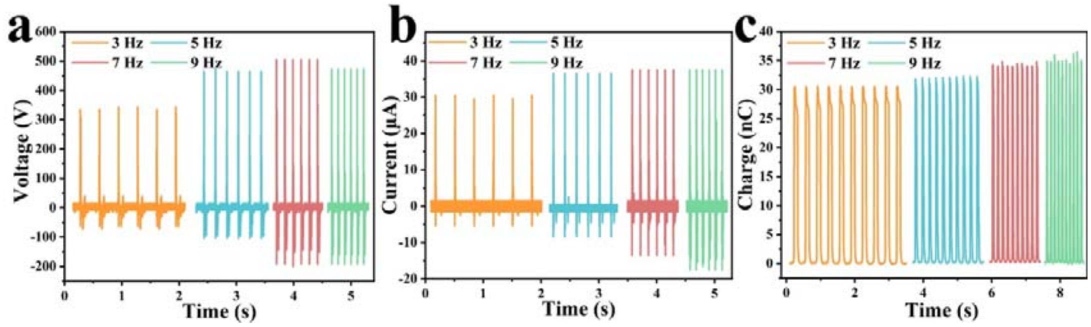

Figure S20. a) Open-circuit voltage, b) short-circuit current, and c) transferred charge of the FG/PPMF TENG in double-electrode mode under various frequency conditions.

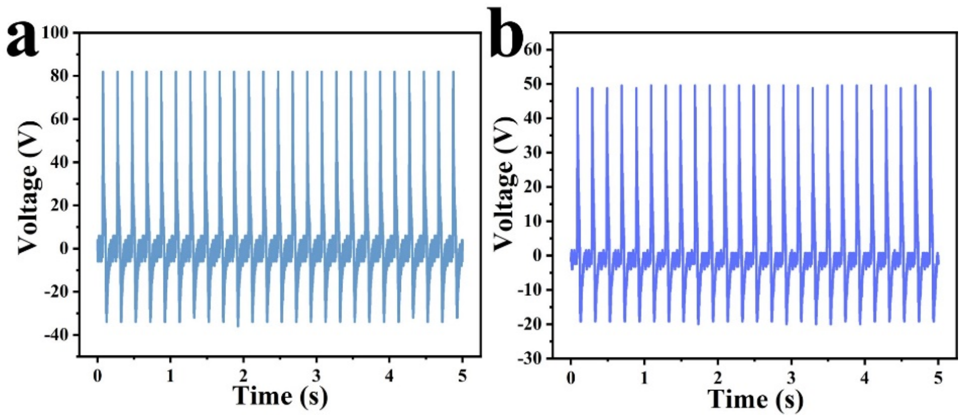

Figure S21. Open circuit voltage of different positive tribomaterials in double-electrode mode at 4.0N, 5Hz. a) Copper sheet as positive tribomaterial. b) PPMF as positive tribomaterial.

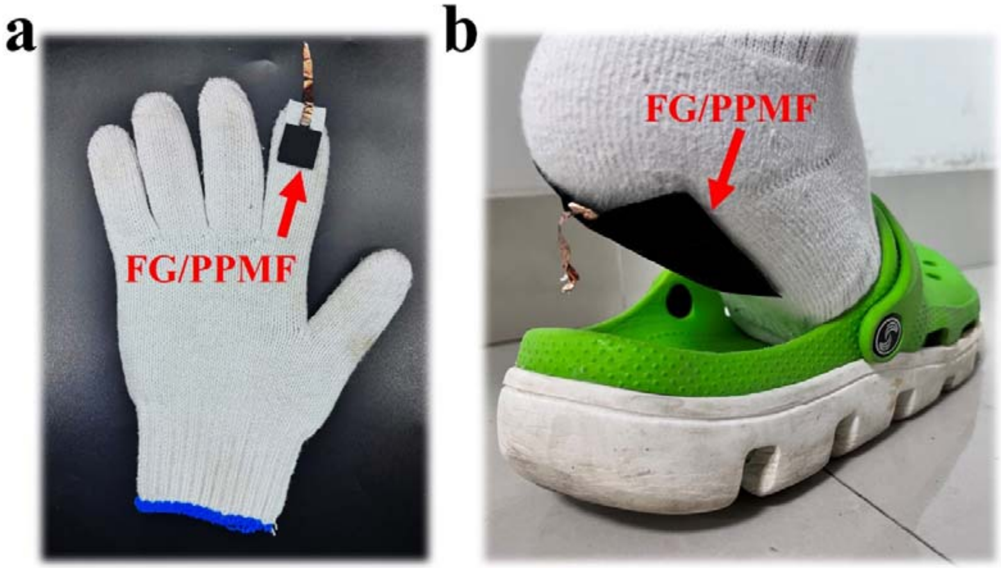

Figure S22. a) Image of the FG/PPMF TENG sensor integrated in glove. b) Image of the FG/PPMF TENG sensor integrated in a sock.

Note S1. Cost analysis of FG/PPMF.

The FG ink obtained through ball milling enabled the preparation of a minimum of 80 FG/PPMF electrodes. The electricity consumed during ball milling amounted to 8.25 kW h, equating to approximately 0.68 dollars. Concurrently, the cost of raw materials necessary for 80 FG/PPMF electrodes was 0.43 dollars. Thus, the average cost per FG/PPMF electrode (2 cm × 2 cm) is 1.39 cents, representing only one-third of the cost of the commercial Ag/AgCl gel electrodes (4.14 cents).

Table S1. Comparison of the sensor's performance with reported research.

Raw material for electrodes	Raw material cost	Preparation method	Customised patterns	Friction and adhesion testing	Washable function	Gas permeability	Conductivity	Appliance	Ref.
PUPDU-Cu/Mxene	High	Spin-coating & Template method & Screen printing	Yes	No	No	-	-	Pressure sensor	[4]
PAN-Au/PA 66-Au	High	Electrospinning & PVD	No	Friction testing	No	-	-	TENG	[5]
TPU/CS/MXene/AgNW	High	Electrospinning & vacuum filtration.	No	No	No	~0.1g cm^{-2} d^{-1}	-	Strain sensor	[6]
MXene/AgNW nonwoven fabrics	High	Screen printing & Spraying	Yes	No	No	~607±8.4mm s^{-1}	~100 Ω/□	Pressure sensor & Humidity sensor	[7]
PAAND/SBS/EGaIn	High	Electrospinning & Screen printing	Yes	No	No	~852 g m^{-2} day^{-1}	~27000 S cm^{-1}	Bioelectrode& Strain sensor	[8]
PDMS/CNT/CB	Middle	Magnetic attraction	No	No	No	~6.7397mg cm^{-2} h^{-1}	-	Pressure sensor	[9]
TPU/Graphite	Low	Fluid dynamics process & Screen printing	Yes	No	No	-	433 S m^{-1}	Na^{+} sensor	[10]
rGO/AgNW	High	Spraying	Yes	No	Yes	-	~20 Ω	Strain sensor	[11]
LMPs@TPU	High	Electrospinning & Electrostatic spraying	No	No	No	-	~38.6 Ω	Strain sensor &Bioelectrode& Non-contact sensor	[12]
Expanded graphite/SG/HDI/ Commercial Fabrics	Low	Dye textile	Yes	No	Yes	~0.35 kg m^{-2} h^{-1}	~15 S cm^{-1}	Strain sensor &Bioelectrode	[13]
MS/rGO/PDMS/ Commercial Fabrics	Middle	Dye textile	No	No	No	~13.6 mg cm^{-2} h^{-1}	~110 kΩ	Strain sensor	[14]
h-WO_3/PET	Middle	Screen printing	Yes	No	No	-	~10^2 kΩ-10^5 kΩ	Humidity sensor	[15]
SEBS-g-MA/b-PEI/Au	High	Sputtering	No	Adhesion testing	No	~70 g m^{-2} h^{-1}	~10 kΩ	Bioelectrode	[16]
Mxene/Cellulose film	High	Immersing	No	No	No	-	10 Ω/sq	Bioelectrode	[17]
Flash graphene/PP nonwoven fabrics	Low	Ball milling & Screen printing	Yes	Yes	Yes	~10.08 mg cm^{-2} h^{-1}	125.2 ± 4.7 Ω/□	Bioelectrode & TENG	This work

Reference:

[1] M. Alegre, I. A. G. Gurtubay, A. Labarga, J. Iriarte, A. Malanda, J. Artieda, *Neuroreport* **2003**, *14*, 381.

[2] E. Liebenthal, M. L. Ellingson, M. V. Spanaki, T. E. Prieto, K. M. Ropella, J. R. Binder, *Neuroimage* **2003**, *19*, 1395.

[3] Q. Li, G. Chen, Y. Cui, S. Ji, Z. Liu, C. Wan, Y. Liu, Y. Lu, C. Wang, N. Zhang, Y. Cheng, K.-Q. Zhang, X. Chen, *ACS Nano* **2021**, *15*, 9955.

[4] K. Liu, M. Wang, C. Huang, Y. Yuan, Y. Ning, L. Zhang, P. Wan, *Advanced Science* **2024**, 11, 2305672.

[5] X. Peng, K. Dong, C. Ning, R. Cheng, J. Yi, Y. Zhang, F. Sheng, Z. Wu, Z. L. Wang, *Adv. Funct. Mater.* **2021**, *31*, 2103559.

[6] M. Chao, P. Di, Y. Yuan, Y. Xu, L. Zhang, P. Wan, *Nano Energy* **2023**, *108*, 108201.

[7] X. Zheng, S. Zhang, M. Zhou, H. Lu, S. Guo, Y. Zhang, C. Li, S. C. Tan, *Adv. Funct. Mater.* **2023**, *33*, 2214880.

[8] F. Chen, Q. Zhuang, Y. Ding, C. Zhang, X. Song, Z. Chen, Y. Zhang, Q. Mei, X. Zhao, Q. Huang, Z. Zheng, *Adv. Mater.* **2023**, *35*, 2305630.

[9] M. Lei, K. Feng, S. Ding, M. Wang, Z. Dai, R. Liu, Y. Gao, Y. Zhou, Q. Xu, B. Zhou, *ACS Nano* **2022**, *16*, 12620.

[10] H. J. Park, J. M. Jeong, S. G. Son, S. J. Kim, M. Lee, H. J. Kim, J. Jeong, S. Y. Hwang, J. Park, Y. Eom, B. G. Choi, *Adv. Funct. Mater.* **2021**, *31*.

[11] T. Kim, C. Park, E. P. Samuel, S. An, A. Aldalbahi, F. Alotaibi, A. L. Yarin, S. S. Yoon, *ACS Appl. Mater. Interfaces.* **2021**, *13*, 10013.

[12] G. Ye, T. Jin, X. Wang, Y. Chen, Q. Wu, Y. Wan, P. Yang, *Nano Energy* **2023**, *113*.

[13] X. Liang, M. Zhu, H. Li, J. Dou, M. Jian, K. Xia, S. Li, Y. Zhang, *Adv. Funct. Mater.* **2022**, *32*, 2200162.

[14] S. Wang, H. Huang, C. Liu, Y. Xia, C. Ye, Z. Luo, C. Cai, C. Wang, L. Lyu, H. Bi, X. Wu, L. Sun, *Adv. Mater. Technol.* **2022**, *7*.

[15] P. Guo, B. Tian, J. Liang, X. Yang, G. Tang, Q. Li, Q. Liu, K. Zheng, X. Chen, W. Wu, *Adv. Mater.* **2023**, *35*.

[16] J. Oh, S. G. Jang, S. Moon, J. Kim, H. K. Park, H. S. Kim, S. M. Park, U. Jeong, *Adv. Healthc. Mater.* **2022**, *11*, 2102703.

[17] D. Song, G. Ye, Y. Zhao, Y. Zhang, X. Hou, N. Liu, *ACS Nano* **2022**, *16*, 17168.
